# Individual behavioral and neurochemical markers of unadapted decision-making processes in healthy inbred mice

**DOI:** 10.1007/s00429-016-1192-2

**Published:** 2016-02-10

**Authors:** Elsa Pittaras, Jacques Callebert, Mounir Chennaoui, Arnaud Rabat, Sylvie Granon

**Affiliations:** 1 Unité fatigue et vigilance, Institut de Recherche Biomédicale des Armées, 91223, Brétigny sur Orge. Equipe d’accueil ‘VIgilance Fatigue et SOMmeil’ (VIFASOM) EA 7330, Université Paris 5 Descartes, 75005 Paris, France; 2 Equipe ‘Neurobiologie de la prise de décision’, Neuro-PSI, CNRS UMR 9197, 91400 Orsay, France; 3 Servie de Biochimie et Biologie Moléculaire, Hôpital Lariboisière, 75010 Paris, France

**Keywords:** Decision-making, Inter-individual differences, Neurobiological markers, Prefrontal, Cortex, Dopamine, Serotonin, Noradrenaline, Flexibility, Safe behavior, Risk-taking

## Abstract

One of the hallmarks of decision-making processes is the inter-individual variability between healthy subjects. These behavioral patterns could constitute risk factors for the development of psychiatric disorders. Therefore, finding predictive markers of safe or risky decision-making is an important challenge for psychiatry research. We set up a mouse gambling task (MGT)—adapted from the human Iowa gambling task with uncertain contingencies between response and outcome that furthermore enables the emergence of inter-individual differences. Mice (*n* = 54) were further individually characterized for locomotive, emotional and cognitive behavior. Individual basal rates of monoamines and brain activation after the MGT were assessed in brain regions related to reward, emotion or cognition. In a large healthy mice population, 44 % showed a balanced strategy with limited risk-taking and flexible choices, 29 % showed a safe but rigid strategy, while 27 % adopted risky behavior. Risky mice took also more risks in other apparatus behavioral devices and were less sensitive to reward. No difference existed between groups regarding anxiety, working memory, locomotion and impulsivity. Safe/rigid mice exhibited a hypoactivation of prefrontal subareas, a high level of serotonin in the orbitofrontal cortex combined with a low level of dopamine in the putamen that predicted the emergence of rigid behavior. By contrast, high levels of dopamine, serotonin and noradrenalin in the hippocampus predicted the emergence of more exploratory and risky behaviors. The coping of C57bl/6J mice in MGT enables the determination of extreme patterns of choices either safe/rigid or risky/flexible, related to specific neurochemical and behavioral markers.

**Electronic supplementary material:**

The online version of this article (doi:10.1007/s00429-016-1192-2) contains supplementary material, which is available to authorized users.

## Introduction

Decision-making is a cognitive process which consists of choosing one option among several alternatives. It progresses from the exploration of unknown options to the exploitation of preferred ones (de Visser et al. 2011a, b, c). During this cognitive process, the decision maker evaluates the value of each option regarding his/her own preferences and the probability to get it which will bring him/her to choose one strategy instead of another one. Such strategies are featured in the Iowa gambling task (IGT) (Bechara et al. 1994), a decision-making task that mimics real life situations by reproducing uncertain conditions based on probabilistic rewards or penalties (Bechara et al. 1994). During this task, subjects have to implicitly discover over time which option is advantageous in the long term, with the discovery that these options are not available under fixed and predictable contingencies. Two categories of behaviors are usually observed: a main one which consists of choosing advantageous options in the long term, and less frequent ones which do not (Bechara et al. 1999, 2002). Using a variant version of the IGT in a healthy population, Bechara et al. (2001, 2002) evidenced the existence of extreme strategies and of a Gaussian distribution of performance.

One of these two extreme strategies observed in a small proportion of healthy subjects is often reinforced in some psychopathological situations in which alteration of prefrontal networks is a hallmark, such as schizophrenia (Brown et al. 2015), depression (Cella et al. 2010), pathological gambling (Clark et al. 2013), or addiction (Balconi and Finocchiaro 2015). Furthermore, adolescents with disruptive behavior disorders and vulnerability for addiction more frequently show risky decision-making (Schutter et al. 2011) and addicted adult patients are more focused on reward which changes their internal state and inner sensation (Paulus and Stewart 2014). It has also been shown that anxious subjects are more likely to focus on internal body-centered cues than on environmental cues (Galván and Peris 2014) and thus are less likely to adapt to changing environments (Robinson et al. 2015). Altogether, it suggests that inter-individual traits are associated to specific strategies during decision-making tasks likely mediated by defective prefrontal cortex activation and/or defective monoaminergic innervations.

Decision-making processes require coordinated activity of multiple brain networks, especially those involving the prefrontal cortex (PFC) (Li et al. 2010). Furthermore, interaction of a limbic loop (affective/emotion) and a cognitive loop (executive/motor) is necessary for adapted decision-making (de Visser et al. 2011a, b, c; Koot et al. 2013). In case of loss after high risk choice, healthy subjects exhibit enhanced PFC activation, whereas anxious subjects exhibit enhanced activation of amygdala and insula (Van den Bos et al. 2013). In addition, prefrontal dopamine levels depend on the emotional content of the decision-making task (Parasuraman et al. 2012) and dopamine transmission modulates the response of the regions of the brain involved in the anticipation and reception of rewards (Dreher et al. 2009). The COMT (catechol-*O*-methyltransferase) gene polymorphism leading to an increased level of endogenous dopamine, and serotonin transporter (5-HTTLPR) polymorphisms have been associated to decision-making impairments (Heitland et al. 2012; Homberg et al. 2008; Malloy-Diniz et al. 2013). However, the results concerning 5-HT are somewhat contradictory (Gendle and Golding 2010; Heitland et al. 2012; Homberg et al. 2008; Koot et al. 2012; Lage et al. 2011; Macoveanu et al. 2013; Pittaras et al. 2013; Stoltenberg et al. 2011; Zeeb et al. 2009).

Several authors adapted the IGT in rodents (van den Bos et al. 2014) to study sex differences (van den Bos et al. 2012), neurobiological substrates (de Visser et al. 2011a, b; Fitoussi et al. 2014; Homberg et al. 2008; Koot et al. 2012; Pais-Vieira et al. 2009; Peña-Oliver et al. 2014; Pittaras et al. 2013; Rivalan et al. 2013; Van Enkhuizen et al. 2013; Zeeb et al. 2009; Zeeb and Winstanley 2011) and environmental (Koot et al. 2013; Van Hasselt et al. 2012; Zeeb et al. 2013) or physiological features (de Visser et al. 2011a; Koot et al. 2012; Pais-Vieira et al. 2009) of decision-making processes. So far, the existence of inter-individual differences in decision-making has been linked to specific behaviors (Rivalan et al. 2009, 2013) and differential neuronal activation (Fitoussi et al. 2014; Rivalan et al. 2009).

As C57BL/6J mice are largely used in neurobehavioral studies worldwide, studying various features of their inter-individual variability could bring novel insight into their cognitive performance in general. These mice are genetically homogeneous, so finding neurobiological markers matching individual profiles is expected to provide robust bases for the emergence of different strategies during decision making, and eventually understanding which regional neurochemical lever could play on these individual traits of behavioral maladjustment. Moreover, we provide here for the first time another way of considering individual strategies during decision-making.

## Materials and methods

### Animals

- 56 C57BL/6J male mice were used for Mouse Gambling Task-MGT, behavioral subsequent analyses and the measurements of brain monoamine levels;
- 30 additional C57BL/6J male mice were used for the c-fos immunochemistry following MGT.

#### Animal housing

Male C57Bl/6J mice bred in Charles’ River facilities (Orleans, France) 5 months old at the beginning of the experiments were used. Mice were housed in a collective cage of three or four in a temperature controlled room (22 ± 2 °C) with a fixed light/dark cycle (light on at 8:00 a.m. and light off at 8:00 p.m.). All experiments were performed during the light cycle between 9:00 a.m. and 5:30 p.m. According to the experiments mice could be food deprived (maintenance at 85 % of the free feeding weight) and always received water ad libitum.

#### Ethics statement

Animals were treated according to the ethical standards defined by the National Center of the Scientific Research for animal health and care with strict compliance with the EEC recommendations (no. 86/609). Ethic protocol number was 2015_04. Moreover, experiments were always done by confirmed experimenters or with their help. Inter-individual studies require large numbers of animals. Despite this difficulty we tried to use as few animals as possible.

### Behavioral procedures

Half of the animals were subjected to the MGT first and then to all other behavioral tests in similar order (novelty exploration, dark-light box, emergence test, working memory, elevated-plus maze, delay-reward task and sucrose consumption), while the second group was subjected first to all behavioral tasks (except elevated plus maze, delay reward, and sucrose consumption that were conducted systematically at the end) and then to the MGT.

#### The mouse gambling task (MGT)

As describe in more details previously (Pittaras et al. 2013) before starting the mouse gambling task mice were habituated to food pellets in operant chambers by doing a nose poke in one illuminated hole to have one food pellet (Supplementary material).

**Fig. 1:**
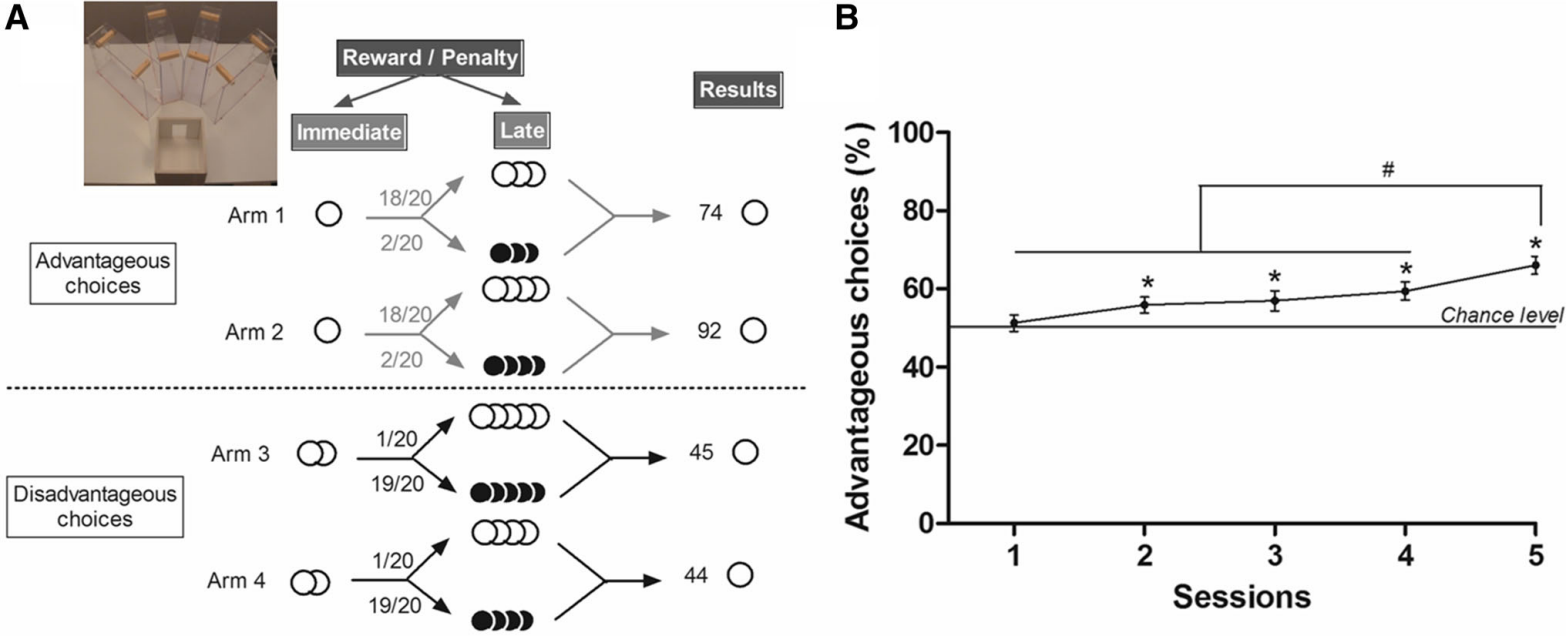
**a** Schematic representation of the MGT experimental design and picture of the maze. *White circle* represented food pellets and *black circle* quinine pellets. Advantageous choices gave access to one food pellet and then to three or four food pellets (18/20) or quinine pellets (2/20). Disadvantageous choices gave access to two food pellets and then to four or five food pellets (1/20) or quinine pellets (19/20). We distinguished advantageous choices from disadvantageous ones because mice earned more pellets (74 or 92 pellets vs. 45 or 44 pellets) after 20 trials by choosing the advantageous ones. **b** Overall percentage (*n* = 54) of advantageous choices (mean ± SEM) for each daily session (1–5). Percentage of advantageous choices at session 5 differed from the other four sessions (*W*, ^#^ *p* < 0.05) and advantageous choices differed from chance level from session 2 to session 5 (*W*, *p < 0.05)

The task took place in a maze with four transparent arms (20 cm long × 10 cm wide) containing an opaque start box (20 cm × 20 cm) and a choice area (Fig. 1a). We used standard food pellets as a reward (dustless precision pellets, grain-based, 20 mg, BioServ^®^, NJ) and food pellets previously steep in a 180 mM solution of quinine as penalty (Van den Bos et al. 2006). The quinine pellets were unpalatable but not inedible. The quality of reward was assured by leaving the mice starving.

There were four different arms: two that gave access to long-term “advantageous” choices and others that gave access to long-term “disadvantageous” choices. In the long-term advantageous arms mice could find one pellet (small reward, as the $50 in the IGT) before a bottle cap containing three or four food pellets on 18 trials over 20 and the same number of quinine pellets for two remaining trials. In the disadvantageous arms mice could find two food pellets (large reward, as the $100 in the IGT) before a bottle cap containing four or five quinine pellets in 19 trials over 20 and the same number of food pellets on the remaining trials (Fig. 1a). Advantageous choices are at first less attractive because of the small immediate reward (one pellet), whereas disadvantageous choices are more attractive at first due to the access to a large immediate reward (two pellets). Despite their immediate reduced attractiveness advantageous choices are advantageous in the long term because animals more often found food pellets and less often the quinine pellets. Conversely disadvantageous choices are less advantageous in the long term because animals more often found quinine pellets than the food pellets (Fig. 1a). Mice therefore had thus to favor the small immediate reward (advantageous choices) to obtain the highest amount of pellets as possible at the end of the session.

During the first session animals were put into the maze for 5 min with food pellets scattered everywhere (habituation). If mice did not eat any food pellets during the first habituation a second 5 min habituation period was conducted. For the following sessions, habituation lasted only 2 min without food pellets available. At the beginning of each trial the mouse was placed in an opaque tube in the starting box to avoid directing the future choice of the animal. After about 5 s, we removed the opaque tube and let the animal free to choose one arm of the maze. Each mouse performed 10 trials in the morning and 10 trials in the afternoon for 5 days (i.e. 5 sessions for a total of 100 trials at the end of the experiment as for the human task (Bechara et al. 1994). Between each trial the maze was cleaned up with distilled water and between each mouse it was cleaned up with a water solution with 10 % of alcohol solution. Localization of advantageous and disadvantageous arms was randomized.

We scored the arm chosen (when the animal crossed 1/3 of the arm) and the food pellet consumption (pellets earned), the number of quinine obtained (but not eaten). A rigidity score was calculated: we measured how many times the animal had chosen the same arm without taking into account the switch between arms. For example, the rigidity score was 25 % if animals chose as many of the advantageous options as the disadvantageous ones. A 50 % score reflected that animal have chosen twice more one arm than the others and a 75 % score that animal have chosen 3/4 one arms than the other. We also measured the number of arms switches between trials.

#### Anxiety and risk-taking (Elevated Plus Maze or EPM)

Mice were tested for their general risk-taking and anxiety behavior with the elevated plus maze (EPM) (Pellow and File 1986), providing an indication of anxiety-like behavior. EPM is an elevated maze composed of two open arms (30 × 5 cm) and two wall enclosed arms (30 × 5 × 25 cm) connected by a central platform (5 × 5 cm). Light intensity on open arms adjusted to 120 lux. The apparatus was elevated 75 cm above the floor. Behavioral testing was started by placing a mouse in the central area facing a closed and an open arm. Exploratory behavior was monitored by a video motility system (Video track, Viewpoint, France) quantified and stored on PC over a period of 5 min.

Parameters for behavioral analyses were: percentage of time spent in open arms (related to total recording time) and head dipping in open arms (as a measure of anxiety and risk-taken, respectively). Visit of an open arm was considered as soon as the mouse placed its two forepaws in the arm. Head dipping were measured manually off line, as the number of time mice bend over the border of the open arms.

#### Sensitivity to the reward task

The sucrose preference was measured as an index for individual sensitivity to reward (Ping et al. 2012) and depression like behavior. Animals were isolated 2 weeks before and during the experiment to have an exclusive access to the two bottles in their home cage. One bottle contained water and the other 1 % solution of sucrose. The consumption of each bottle was measured by weighting bottles every day at the same hour.

As sucrose solution is new and could be a stressor for mice on day 1 animals had only sucrose available in the two bottles. Days 2 and 3, animals had one bottle of water and one of sucrose but the place of the two bottles was exchanged between day 2 and 3. We measured a sucrose preference score as follows: $[ ( {\text{sucrose consumption)/(sucrose}} +\, {\text{water consumption)]}}\; \times \; 100.$

#### Delay reward task

The behavioral procedure was adapted from a previous work (Serreau et al. 2011). Operant chambers contained two holes for nose poke. During the training phase (9 days), making a nose poke in one of the two holes (“small and immediate reward” hole, H1) led to the delivery of one food pellet (dustless precision pellets, grain-based, 14 mg, BioServ^®^, NJ). A nose poke in the other hole (“large and delayed reward” hole, H4) resulted in the delivery of four food pellets. The house light remained on until the animals visited the food magazine and was switched off after 20 s. During the test session (five consecutive days) an additional delay was inserted between a nosepoke in the H4 hole and the delivery of the pellets. The delay remained the same during the entire daily session and increased every day (0, 10, 30, 50, 90 s).

A shift in the choices from the hole that gives high rewards to the hole that gives low rewards as a function of the delay before food delivery is taken as an index of the ability to wait for a larger reward and to control the frustration imposed by the delay (Serreau et al. 2011). The percentage of H4 choices during each session was scored.

#### Novelty exploration

Novelty exploration was realized in a transparent empty Plexiglas cage. We measured the mice locomotor activity and exploration (Supplementary methods).

#### Anxiety tasks (emergence, dark-light)

##### Emergence task

Emergence task was done in a large white openfield connected to a small black box protected from light. We recorded on line: the time took by the mouse to emerge in the openfield and the percentage of time spent in the openfield (Supplementary methods).

##### Dark-light task

Dark-light task was done in an apparatus composed of two boxes: one black box protected from light by a cover and the other one white and brightly illuminated. Behavioral measures were: initial latency to escape the light box, number of mice passing from the light box to the dark box and the percentage of total time spent in the light box (Supplementary methods).

#### Working memory task (T-maze)

The behavioral task used to test working memory is based on spontaneous alternation (SA) behavior (Piérard et al. 2006). This task was carried out in a T-maze made of opaque grey Plexiglas. We measured the spontaneous alternation with a 30 s inter-trial interval (ITI) (Supplementary methods).

### c-fos immunohistochemistry

24 mice were trained in the MGT protocol before killing: habituation in operant chambers for 2 weeks and 1 week of MGT. As a control, six mice were subjected to similar initial training and then to a variant of the MGT in which mice did not have to choose between arms with food available everywhere in the maze.

#### Killing and sampling

Animals were anesthetized (for 2 ml: 50 μL of Rompun 2 %; 600 μL of ketamine 500; 1350 μL PBS 1×—1 mL for 10 g) 90 min after the end of the last MGT session. This timing allows the synthesis of c-fos (early immediate gene) protein in the nuclei of activated neurons (Chauveau et al. 2014). Control mice were also anesthetized the fifth day with the same timing as MGT mice.

Mice were immediately perfused transcardially with 20 mL phosphate-buffered saline (PBS) and then with 50 mL of 4 % paraformaldehyde (PFA). Brains were removed, fixed during 24 h with PFA and cryoprotected with increased sucrose solution for 3 days at 4 °C. Brains were thereafter put at −20 °C in glycerol before immunological experiments.

#### Immunohistochemistry

Brains were sliced with a vibratome (Leica, VT1000E) on a coronal plane into 40 µm sections. Immunochemistry began with two 4 × 10 min rinses in PBS. Then endogenous peroxidases were neutralized for 30 min in PBS containing 3 % H_2_O_2_. To block the nonspecific site, we used PBS solution with 1 % bovine serum albumin (BSA), 3 % normal goat serum (NGS) and 0.2 % Triton X-100 for 2 h. c-fos immunolabeling was performed with a purified polyclonal rabbit IgG anti-human c-fos [anti c-fos (Ab-5)(4-17) rabbit pAb, CALBIOCHEM] diluted 1:20,000 in 1 % BSA, 3 % NGS and 0.2 % Triton X-100 for 38 h. After 4 × 10 min rinses in PBS, sections were incubated for 2 h with secondary biotinylated antibody [biotin goat anti-rabbit IgG (H+L), INTERCHIM] diluted 1:2,000,000 in 1 % BSA, 3 % NGS and 0.2 % Triton X-100). After 4 × 10 min rinses in PBS, the staining was revealed using H_2_O_2_ and diaminobenzidine (D-5905, SIGMA) for 3 min. After rinsing, sections were flattened on SuperFrost glass slides (Menzel-Gläser, Braunschweig, Germany), dehydrated with xylene and mounted with Eukitt solution.

#### Quantification of c-fos positive (c-fos^+^) nuclei

Quantification was performed by identifying spot positions. c-fos^+^ nuclei were counted with ICY software (http://icy.bioimageanalysis.org/) after acquiring images using a digital camera (Nikon DXM 1200) of an Olympus BX600 microscope coupled to software (Mercator Pro; Explora Nova, La Rochelle, France). The constant use of a 10 × Plan Apo objective allowed us to have good resolution for c-fos immunochemistry. The focus was set on the upper face of each section before digitization. Each region of interest (ROI) was delimited on the screen for each picture based on the mouse atlas (Paxinos and Franklin 2001). ICY software directly counts the number of cells in the ROI. The density of cell per square micrometer was calculated after and normalized in relation to the control. The ROI chosen included cortical areas known to be involved in decision making as well as other brain areas know to be involved in novelty, exploration, reward and motivation (Avale et al. 2011): prelimbic (PrL), infralimbic (IL), orbitofrontal lateral, median, dorsolateral and ventral cortex (OFC), nucleus accumbens (NAcc), caudate putamen (CPu), basolateral amygdala (BLA), basomedian amygdala (Amy), hippocampus (H), motor cortex (M), cingular cortex (Cg) and agranular and granular insular cortex, dorsal and ventral (CIns). Figures 7, 8 and 9 from the atlas were chosen to analyze PrL and OFC. Figures 17, 18 and 19 were chosen to analyze PrL, IL, Cg, M, CIns, NAcc, CPu and Figs. 41, 42 and 43 to analyze BLA, Amy and H (Paxinos et al. 2001).

### Basal monoamine brain level analysis

#### Brain extraction

Brains were removed at least 1 month after the last behavior task. Animals were slightly anesthetized with Isoflurane (Iso-Vet, 1000 mg/g) before cervical dislocation. Brains were rapidly removed and stored at −80 °C.

#### Brain section and punch

Brains were placed at −20 °C the day before slicing. One hour before slicing, brains were brought to the cryostat and maintained at −13 °C. Coronal sections (140 μm) were performed on the cryostat. The punches (diameter 0.75 mm) of each brain region were precisely localized and punched using the mouse atlas (Paxinos et al. 2001).

As shown in supplementary information, we punched in regions of interest from both hemisphere: orbitofrontal cortex (OFC) (lateral, median, dorsolateral and ventral), prelimbic (PrL), insular cortex (CIns) (agranular and granular insular cortex, dorsal and ventral), nucleus accumbens (NAcc) (core and shell), the amygdala (Amy) (basolateral amygdala and amygdalian nucleus), the hippocampus (H) and the caudate putamen (CPu) (primary and secondary) (Fig. S1).

#### HPLC dosage

Amount of dopamine (DA), serotonin (5-HT) and noradrenaline (NA) was quantified using high performance liquid chromatography (HPLC) techniques.

Prior to analysis, brain tissues were crushed in 350 μL of 0.2 M perchloric acid and centrifuged at 22,000*g* for 20 min at 4 °C. The supernatants were collected and filtered through a 10 kDa membrane (Nanosep, Pall) by centrifugation at 7000*g*. Then, a 20 μL aliquot of each sample was analyzed for 5-HT by fluorometric detection (Kema). The amounts of catecholamines (dopamine and noradrenaline) were measured by electrochemical detection on a serial array of coulometric flow-through graphite electrodes (CoulArray, ESA) (Gamache). Analysis, data reduction, and peak identification were fully automated. Results were expressed as fentomoles/milligram of fresh tissues (Gamache et al. 1993; Kema et al. 1993).

### Statistical analysis

#### Sub-group formation

To distribute animals among groups regarding their performances we calculated the mean of 30 last trials (i.e. when performances was stable) and used a *k*-mean clustering separation with Statistica^®^ software (version 12) (Timmerman et al. 2013), so that animal belonged to a set that had the closest mean to its own performance value. Three groups were defined: animals which chose mostly advantageous options at the end of the experiment, thereafter called “safe” group, animals which explored the different options at the end of the experiment, thereafter called “risky”, and animals which exhibited an intermediate behavior and distributed their choices between sporadic risky choices and high proportion of advantageous choices, thereafter called “average”.

#### For a group size exceeding 30 animals

To compare global performances in the MGT and the global differences from chance level (50 %), we used a Student’s test with Bonferroni correction. Repeated ANOVAs (main factors were group and sessions) followed by post hoc analysis (student tests) when appropriate were conducted to see assess evolution of performances with time. Correlation was carried out using Spearman correlation (*S*). The statistical significance threshold was set at *p* < 0.05.

#### For group size less than 30 animals

We used non-parametric statistical analyses. To compare global performances evolution (differences between sessions) in the MGT and the global differences from chance level (50 %), we used a Wilcoxon test (*W*). To analyze differences between the three groups of performance (choices and pellets consumption) we used a Kruskal–Wallis (KW). To further show group differences two by two we used Mann–Whitney (MW). Non-parametric statistical tests mentioned above were used for all data (behavioural, c-fos and neurochemical measures). Correlation was carried out using Pearson correlation (*P*). The statistical significance threshold was set at *p* < 0.05.

## Results

### Mouse gambling task (MGT)

#### Overall performances

Two mice were excluded from the study because of a spatial bias. As illustrated on Fig. 1b mice initially chose the two options equally on the first session (51.2 ± 2 %) (*t* test; Bonferroni *p* < 0.01: *t* = 0.619; *p* = 0.5388). Then, and until the end of the task, mice significantly preferred choosing advantageous options (from 55.8 ± 2 to 66 ± 2.3 %) (*t* test Bonferroni *p* < 0.01—session 2: *t* = 2.849, *p* = 0.0062; session 3: *t* = 2.748, *p* = 0.0082; session 4: *t* = 4.142, *p* = 0.0001; session 5: *t* = 6.993, *p* < 0.0001). Over time, mice developed a stable choice preference for advantageous options (*t* test, Bonferroni *p* < 0.005—session 1 vs. session 4: *t* = −3.515, *p* = 0.0009; session 1 vs. session 5: *t* = −5.803, *p* < 0.0001). Moreover, choice preference at session 5 differed from all the other sessions (*t* test Bonferroni *p* < 0.005—session 2 vs. session 5: *t* = −3.938, *p* = 0.0002; session 3 vs. session 5: *t* = −3.203, *p* = 0.0033; session 4 vs. session 5: *t* = −3.209, *p* = 0.0023). These data indicated that mice were able to discriminate long-term advantageous arms from those that would be more advantageous in the short term but not in the long term (also named “disadvantageous”).

#### Inter-individuals’ differences among the inbred performing the MGT

Animals were separated using the *k*-mean statistical method based on their overall preferences for advantageous choices during the last 30 trails (differences from chance level for block of 10 trials: *t* test: trials 1–10: *t* = 0.630, *p* = 0.5314; trials 11–20: *t* = 0.425, *p* = 0.6723; trials 21–30: *t* = 2.043, *p* = 0.0461; trials 31–40: *t* = 2.775, *p* = 0.0076; trials 41–50: *t* = 1.772, *p* = 0.0002; trials 51–60: *t* = 2.951, *p* = 0.0047; trials 61–70: *t* = 2.914, *p* = 0.0052; trials 71–80: *t* = 4.324, *p* < 0.0001; trials 81–90: *t* = 5.105, *p* < 0.0001; trials 91–100: *t* = 7.702, *p* < 0.0001). We have chosen to divide our animals into three groups because of the Gaussian individual repartition (Fig. S2B). 27 % of all animals did not show a significant preference for long-term advantageous options (45 ± 2.8 %) (*W* test—session 3: *Z* = −0.3629, *p* = 0.5294; session 4: *Z* = −1.051, *p* = 0.2934; session 5: *Z* = −1.734, *p* = 0.0830). They constituted the group of “risky” mice. Mice of the “average” group (42 % of the overall group) developed a significant preference for the long term advantageous options (*W* test—session 1: *Z* = −2.500, *p* = 0.0124; session 2: *Z* = −2.972, *p* = 0.003; session 3: *Z* = −2.906, *p* = 0.0037; session 4: *Z* = −3.493, *p* = 0.0005; session 5: *Z* = −4.015, *p* < 0.0001) but they can be statistically distinguished from the last group, the “safe” mice (29 % of the overall group) which strongly developed a preference for advantageous options (*W* test—session 1: *Z* = −0.943, *p* = 0.3454; session 2: *Z* = −2.040, *p* = 0.0413; session 3: *Z* = −2.386, *p* = 0.0171; session 4: *Z* = −3.408, *p* = 0.0007; session 5: *Z* = −3.516, *p* = 0.0004). A two-way ANOVA revealed a significant interaction effects between groups and sessions [*F*(2,4) = 3.744, *p* = 0.0004]. The three groups (safe, average and risky) were significantly different from each others from the 4th session of the task (MW—safe vs. risky; session 4: *U* = 13.500, *p* < 0.0001; session 5: *U* = 0.000, *p* < 0.0001; safe vs. average; session 4: *U* = 50.500, *p* = 0.0001; session 5: *U* = 48.500, *p* < 0.0001; risky vs. average; session 4: *U* = 72.000, *p* = 0.0027; session 5: *U* = 10.000, *p* < 0.0001). These results showed that inter-individual differences existed among inbred mice performing MGT and remained steady.

**Fig. 2:**
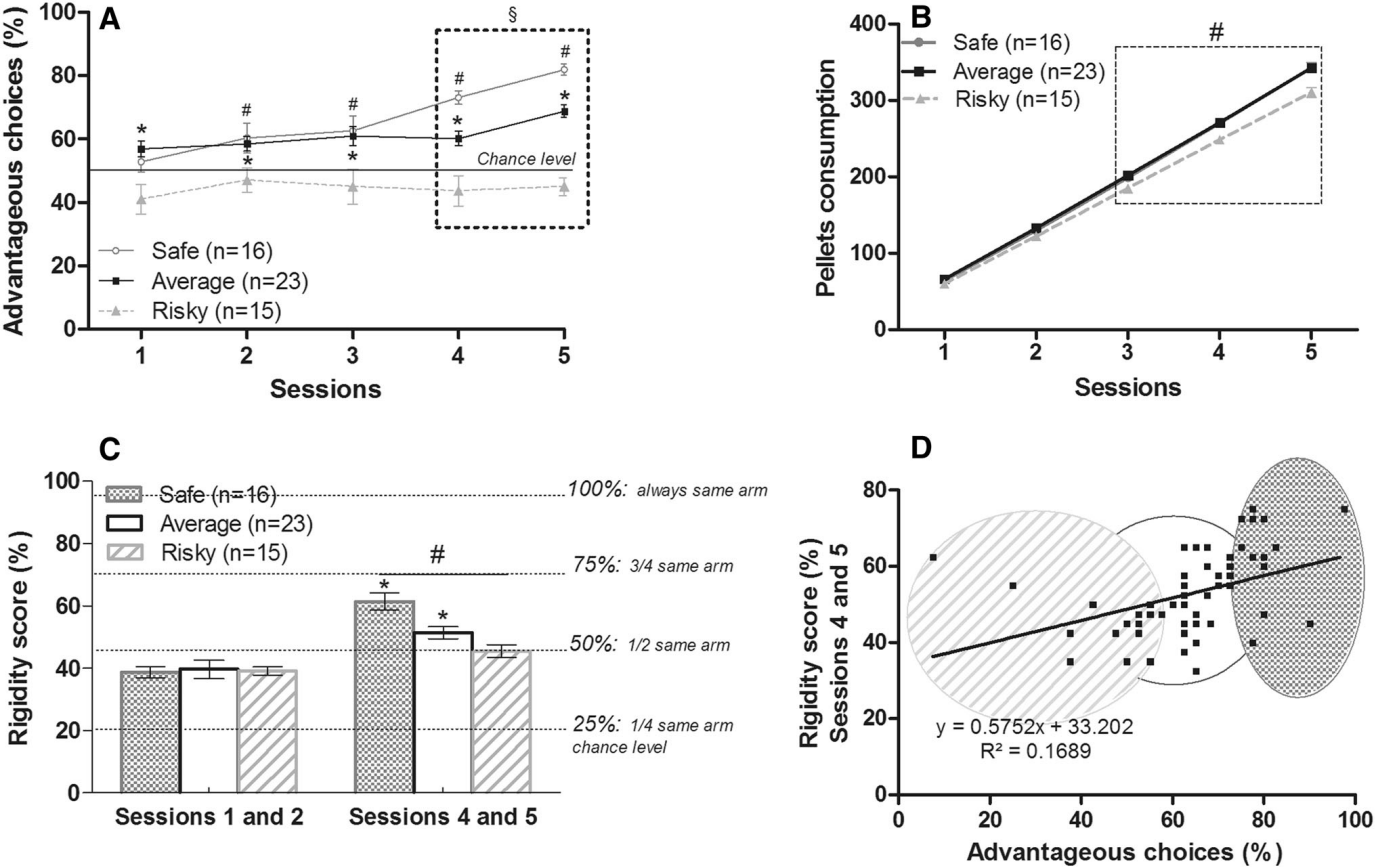
Inter-individual differences that emerged during the MGT. **a** Performances evolution during MGT for safe (*n* = 16, *grey circle*), average (*n* = 23, *black square*) and risky animals (*n* = 15, *grey triangle*). Safe and average groups differed from chance but not risky group (*W* safe, ^#^ *p* < 0.05; average, *p < 0.05). The three sub-groups differed from each other during the two last sessions (MW, ^§^ *p* < 0.05). **b** Cumulative pellet consumption across sessions (addition of pellets obtained from the beginning for each session). Safe and average animals did not differ from each other but the three groups differed the three last sessions (KW, ^#^ *p* < 0.05). **c** Rigidity score was calculated as the percentage of the more chosen arms during the two first sessions and the two last sessions of the task. A 25 % score reflected an equal choice between the 4 arms and a 100 % score reflected a systematic choice of the same arm. Rigidity score of safe and average animals differed between sessions 1 and 2 and sessions 4 and 5 (*W*, *p < 0.05) and the three groups differed from each other during sessions 4 and 5 (KW, ^#^ *p* < 0.05) with safe mice exhibiting more rigid behavior. Animals’ performance during the 30 last trials were correlated with the rigidity score (**d**, *p* < 0.05). Safe animals are grouped in the *darker* *ellipse*, average animals are enclosed in the *white circle*, and risky animals grouped in the *grey stripes*

We observed a significant interaction between sessions and groups for pellets cumulative consumption [repeated measure ANOVA: *F*(2,4) = 8.093; *p* < 0.0001]. As illustrated on Fig. 2b, safe and average mice gained more pellets than risky one at the end of the task (342 pellets for safe and average mice vs. 310 pellets for risky mice) (MW—safe vs. risky; session 1: *U* = 68.000, *p* = 0.0398; session 2: *U* = 83.500, *p* = 0.1491; session 3: *U* = 63.500, *p* = 0.0255; session 4: *U* = 50.000, *p* = 0.0057; session 5: *U* = 41.500, *p* = 0.0019; safe vs. average; session 1: *U* = 151.500, *p* = 0.3534; session 2: *U* = 151.500, *p* = 0.3534; session 3: *U* = 158.500, *p* = 0.4666; session 4: *U* = 176.500, *p* = 0.8304; session 5: *U* = 182.500, *p* = 0.9658; risky vs. average; session 1: *U* = 80.000, *p* = 0.0057; session 2: *U* = 70.000, *p* = 0.0022; session 3: *U* = 56.500, *p* = 0.0005; session 4: *U* = 58.000, *p* = 0.0006; session 5: *U* = 36.500, *p* < 0.0001). The weight of the animals of the three groups did not differ for any daily session (Fig. S2A; KW: session 1: *H* = 5.974; *p* = 0.0504; session 2: *H* = 5.297; *p* = 0.0707; session 3: *H* = 3.559; *p* = 0.1687; session 4: *H* = 5.309; *p* = 0.0703; session 5: *H* = 3.452; *p* = 0.1780) showing that the difference in performance cannot be due to weight differences. Moreover, risky mice obtained (but not ate) more quinine pellets than others mice (Fig. S2D). Therefore, mice strategies for long-term advantageous options led to a larger amount of pellets consumed.

Rigidity score was calculated as the percentage of the more chosen arms during the two first sessions and the two last sessions of MGT. As illustrated in Fig. 2c, rigidity scores were close to 39.1 ± 1 % at the beginning of MGT for all mice and not different among them (MW—two first sessions—safe vs. average: *U* = 172.000, *p* = 0.7319; risky vs. average: *U* = 151.000, *p* = 0.5208; risky vs. safe: *U* = 111.000, *p* = 0.7220). At the end of MGT, only safe and average mice showed a significant increase of their rigidity scores (from 38.75 ± 1.8 to 61.4 ± 2.7 % and from 39.1 ± 1.3 to 51.4 ± 1.9 %; *W* safe *Z* = −3.413, *p* = 0.0006; average *Z* = −3.597, *p* = 0.0003; risky *Z* = −1.433, *p* = 0.1520). Rigidity scores were significantly different among 3 groups at the end of the task (MW—two last sessions—safe vs. average: *U* = 92.500, *p* = 0.009; risky vs. average: *U* = 106.000, *p* = 0.047; risky vs. safe: *U* = 31.500, *p* = 0.0005) and correlated with the percentage of advantageous choices during the 30 last trials (*S* correlation: *r* ^2^ = 0.1689; *p* = 0.001). Moreover, the number of switch between arms was significantly different between the three groups and less important for safe mice (Fig. S2C). Interestingly, a majority of safe mice (68 %) chose the arm 4, when they chose disadvantageous options. This arm was associated in general with less quinine pellets but also less food pellets when an important reward occurred. Moreover, 43 % of safe mice chose more often the arm 2 which is associated generally with more food pellets earned but also more quinine pellets when a penalty occurred. Conversely, 61 % of average mice chose more often the arm 2 and 52 % the arm 4 and 40 % of risky mice chose more often the arm 2 and the arm 4. These data indicated that only risky mice kept a strategy in which they continued to explore all different options (advantageous and disadvantageous options) until the end of MGT despite the less reward obtained (total pellets consumption) and that safe mice adopted a rigid strategy which aimed to obtained less quinine pellets.

#### Behavioral characterization of the three MGT groups

##### Sucrose preference

**Fig. 3:**
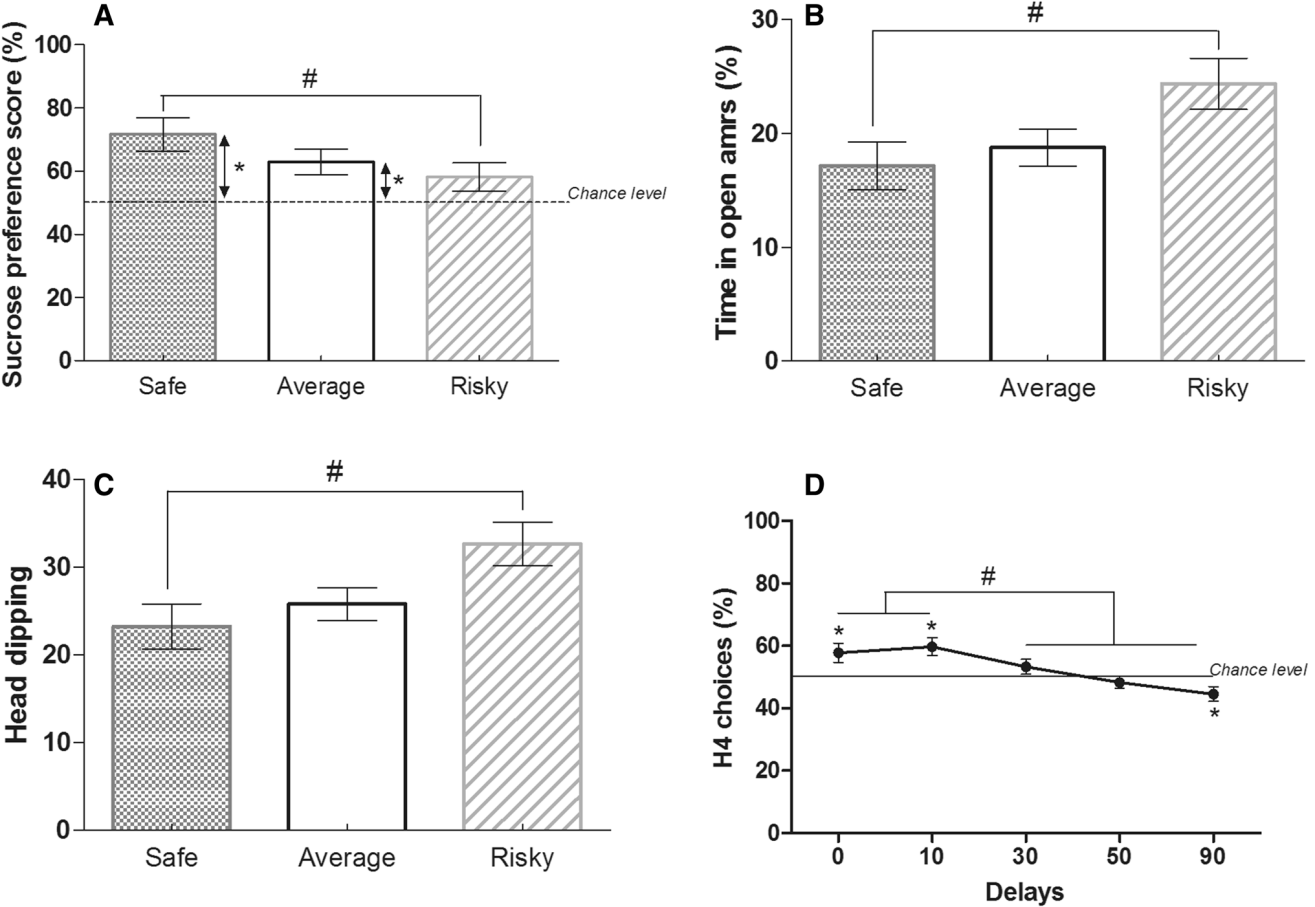
Individual behavioral characterization. **a** During the sucrose preference task, average (*n* = 23) and safe (*n* = 16) animals significantly preferred sucrose over water whereas risky mice (*n* = 14) did not differ from chance (*W*, *p < 0.05). Safe and risky animals differed from each other (MW, ^#^ *p* < 0.05). **b** Risky (*n* = 15) animals spent more time in the open arms of the elevated plus maze and did more head dipping (**c**; MW, ^#^ *p* < 0.05) than average (*n* = 23) and safe (*n* = 16) mice. **d** Percentage of H4 choices during the delay reward task changed across sessions (*W*, differences from chance *p < 0.05; differences between sessions ^#^ *p* < 0.05) but there was no differences between groups and no interaction groups × sessions

Only average (62.8 ± 4 %) and safe (71.6 ± 5.3 %) mice significantly chose more often, and more importantly, the sucrose solution in comparison to water (*W* task—safe *Z* = 4.240, *p* = 0.0007; average *Z* = 3.102, *p* = 0.0022; risky *Z* = 1.851, *p* = 0.1981). Safe mice showed a significantly higher preference for sucrose compared to risky ones (MW: *U* = 63.000; *p* = 0.0417; Fig. 3a).

##### Anxiety like and risk-taking behaviors

Compared to safe mice, risky mice spent significantly more time in open arms (MW: *U* = 62.000; *p* = 0.0219; Fig. 3b) and did more head dipping (MW: *U* = 61.000; *p* = 0.0197) (Fig. 3c).

##### Delay-reward

The percentage of H4 choices (“large and delayed reward” hole) shifted to H1 (“small and immediate reward” hole) when the delay was higher than 30 s (from 57.7 ± 3 to 44.4 ± 2.3 %; Fig. 3d). There was a significant effect of sessions [repeated measurement ANOVA: *F*(4) = 13.742, *p* < 0.0001] but no significant effect for groups [repeated measurement ANOVA: *F*(2) = 0.058, *p* < 0.9435; Fig. 3d] and interaction sessions × groups [repeated measurement ANOVA: *F*(2,4) = 1.026, *p* < 0.4174]. This suggests that all groups exhibited a similar switch from high to low reward as the delay to get the reward increased. Percentage of H4 choices differed from days 1 and 2 to days 3, 4 and 5. These data indicated that the overall switch between high and low reward happened around 30–40 s for all animals, like it was shown before (Serreau et al. 2011). As a result, all animals were able to discriminate a small reward from a large reward and to shift toward large choices when the delay was too long.

##### Control behaviors (Figs. S2, S3)

The three groups of mice (safe, average and risky) did not differ regarding working memory (KW: *H* = 2.009; *p* = 0.3663), anxiety (KW—dark/light—*H* = 1.452; *p* = 0.4837; emergence *H* = 2.637; *p* = 0.2676), locomotor activity (KW: novelty exploration *H* = 2.527; *p* = 0.2826) and exploration (KW: *H* = 1.348; *p* = 0.5097; Figs. S2, S3).

In summary, these behavioral results showed that safe and risky mice have opposite behaviors. Safe mice were able to discriminate a more rewarding solution and took less risk in two different behavioral devices (EPM and MGT). Risky mice were more prone to take risks and less able to discriminate a more rewarding solution.

#### Neurobiological characterization of the three MGT groups

##### c-fos activation induced by MGT

Other mice were used to determine the c-fos network activation after performing MGT. We first confirmed that another group of 24 more mice were able to discriminate long-term advantageous choices from long-term disadvantageous ones. Second, we observed individual differences with three groups of mice (safe, average and risky) based on their behavioral inter-individual differences (Fig. S5).

**Fig. 4:**
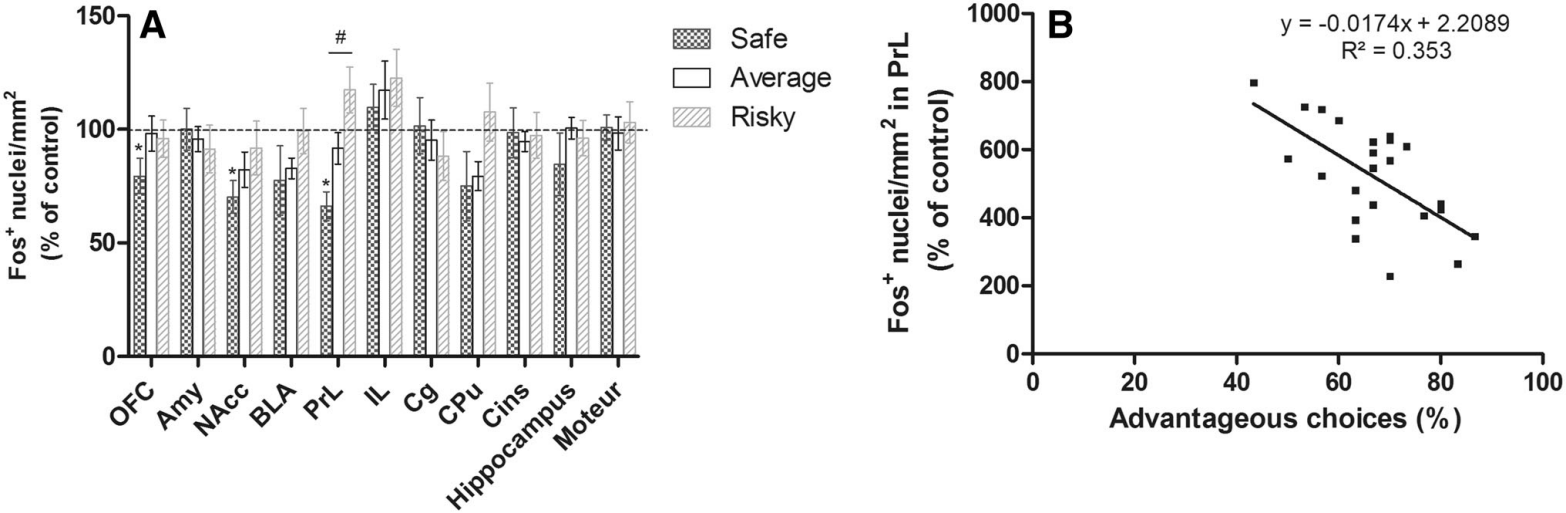
**A.** Relative quantification of fos reactivity (mean ± SEM) in the orbitofrontal cortex (OFC), amygdala (Amy), nucleus accumbens (NAcc), basolateral amygdala (BLA), prelimbic (PrL), infralimbic (IL), cingular cortex (Cg), caudate putamen, (CPu), insular cortex (CIns), hippocampus and motor cortex. c-fos quantification was expressed as a percentage of that measured in the control group (dotted line) for safe (*n* = 5), average (*n* = 13) and risky (*n* = 6) mice. Only safe mice differed from the controls for the OFC, NAcc and PrL (*W*, *p < 0.05). The three groups differed from each other only regarding c-fos activation of the PrL (KW, ^#^ *p* < 0.05). **b** c-fos reactivity was correlated with the percentage of advantageous choices during the 30 last trials (*p* < 0.05)

No differences existed between the three groups regarding the OFC (KW: *H* = 3.510; *p* = 0.1729), Amy (KW: *H* = 0.939; *p* = 0.6253), NAcc (KW: *H* = 4.151; *p* = 0.1255), BLA (KW: *H* = 2.229; *p* = 0.3280), IL (KW: *H* = 0.450; *p* = 0.7985), Cg (KW: *H* = 0.704; *p* = 0.7034), CPu (KW: *H* = 3.723; *p* = 0.1554), CIns (KW: *H* = 2.038; *p* = 0.3609), *H* (KW: *H* = 0.166; *p* = 0.9202) and M (KW: *H* = 0.445; *p* = 0.8006) (Fig. 4a). Activation of c-fos protein was significantly different among three groups in the PrL (KW: *H* = 7.872; *p* = 0.0195) and was correlated with the percentage of advantageous choices during the 30 last trials (*S* correlation: *r* ^2^ = 0.353; *p* = 0.0094, Fig. 4a, b). Interestingly, c-fos protein activity in the PrL was also correlated with the rigidity score of mice during the MGT (data not shown, *y* = −0.104*x* + 59.533, *R* ^2^ = 0.0615; *p* = 0.004). Indeed, c-fos protein activation of safe mice was less important than risky ones in this cortical area (MW: safe vs average *U* = 13.000, *p* = 0.0546; safe vs risky *U* prime = 25.000, *p* = 0.009; risky vs average *U* = 14.000, *p* = 0.0682; Fig. 4).

Safe mice differed from the control mice regarding c-fos activation in the OFC (MW: *U* = 3.000, *p* = 0.0472), NAcc (MW: *U* = 3.000, *p* = 0.0472) and PrL (MW: *U* = 0.000, *p* = 0.0062) and no differences between average or risky compared to the control group (MW: always *p* > 0.05; Fig. 4).

##### Basal rate of cerebral monoamines for the three MGT groups (*n* = 50)

**Fig. 5:**
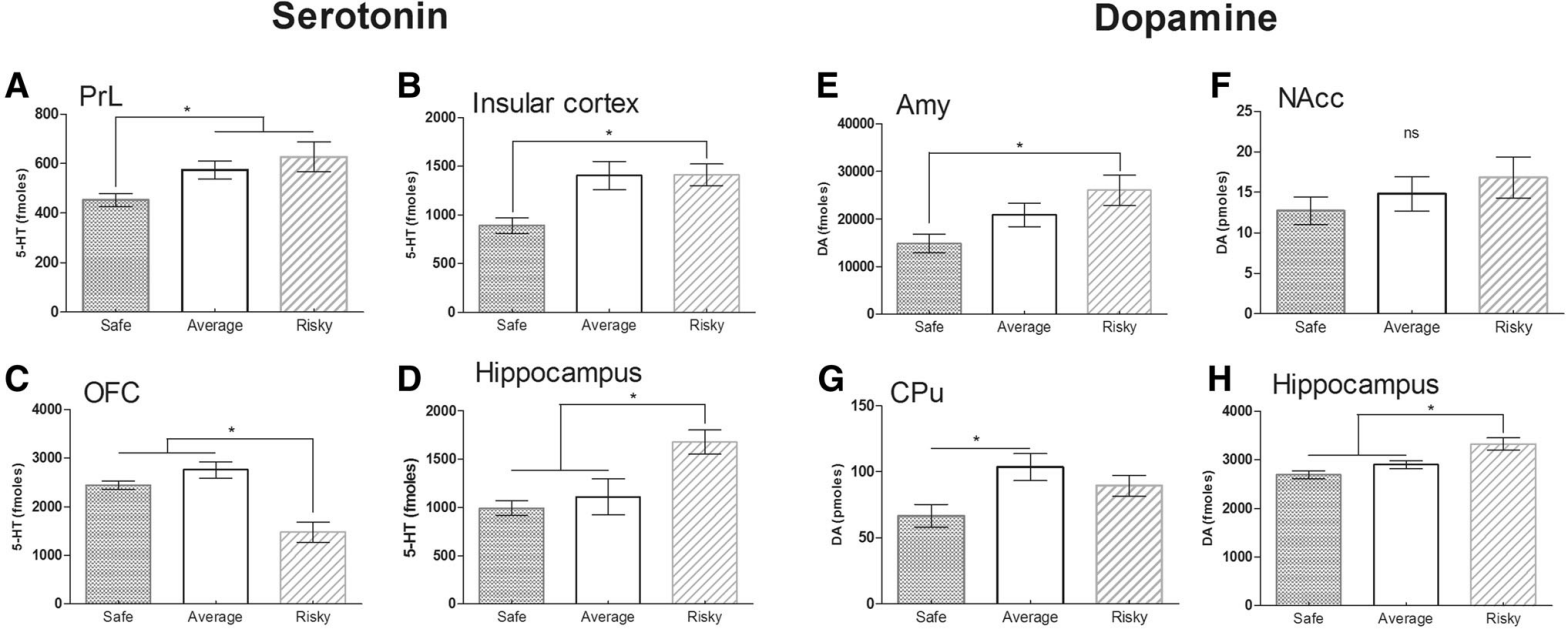
Basal rates of serotonin (5-HT) (**a**–**d**) and dopamine (DA) (**e**–**h**) in the prelimbic (PrL), the insular cortex (CIns), orbitofrontal cortex (OFC), the hippocampus, the amygdala (Amy), the nucleus accumbens (NAcc) and the caudate putamen (CPu) for safe (*n* = 16), average (*n* = 20) and risky (*n* = 14) mice. Results are expressed as mean ± SEM for each group. *p < 0.05 represented a significant difference between each groups (MW). Safe mice had a low level of 5-HT in the PrL, the CIns and less DA in the Amy and the CPu. Risky mice had a low level of 5-HT in the OFC and a higher level in the hippocampus. Risky mice also had a higher level of DA in the hippocampus. No significant difference existed between groups regarding the NAcc (ns)

As a result, risky mice showed a higher level of serotonin (5-HT) (KW: *H* = 17.283; *p* = 0.0002; MW: safe vs. average *U* = 43.000, *p* = 0.3237; safe vs. risky *U* = 21.000, *p* = 0.002; risky vs. average *U* = 43.000, *p* = 0.0007), dopamine (DA) (KW: *H* = 12.048; *p* = 0.0024; MW: safe vs. average *U* = 68.500, *p* = 0.2325; safe vs. risky *U* = 32.000, *p* = 0.0009; risky vs. average *U* = 68.500, *p* = 0.0124) and noradrenaline (NA) (KW: *H* = 14.103; *p* = 0.0009; MW: safe vs. average *U* = 53.000, *p* = 0.2862; safe vs. risky *U* = 29.000, *p* = 0.006; risky vs. average *U* = 55.000, *p* = 0.0029) in the *H* (Figs. 5d, h, S6D).

Safe mice had a lower level of 5-HT than risky ones in the PrL (KW: *H* = 9.691; *p* = 0.0079; MW: safe vs. average *U* = 129.000, *p* = 0.0057; safe vs. risky *U* = 45.500, *p* = 0.0057; risky vs. average *U* = 129.000, *p* = 0.7003), CIns (KW: *H* = 17.047; *p* = 0.0002; MW: safe vs. average *U* = 122.000, *p* = 0.0.0004; safe vs. risky *U* = 27.500, *p* = 0.004; risky vs. average *U* = 122.000, *p* = 0.5288; Fig. 5a,b). Conversely, risky mice had a lower level of 5-HT compared to safe mice in the OFC (KW: *H* = 17.233; *p* = 0.0002; MW: safe vs. average *U* = 34.000, *p* = 0.0856; safe vs. risky *U* = 36.500, *p* = 0.0017; risky vs. average *U* = 34.000, *p* = 0.002; Fig. 5c).

Safe mice had less DA in the Amy (KW: *H* = 7.071; *p* = 0.0291; MW: safe vs. average *U* = 125.000, *p* = 0.1710; safe vs. risky *U* = 125.000, *p* = 0.0053; risky vs. average *U* = 60.000, *p* = 0.2207), CPu (KW: *H* = 7.270; *p* = 0.0264; MW: safe vs. average *U* = 110.000, *p* = 0.013; safe vs. risky *U* = 67.000, *p* = 0.0614; risky vs. average *U* = 110.000, *p* = 0.2938; Fig. 5e, g) and no differences existed between groups regarding the NAcc and OFC (KW: *H* = 1.519; *p* = 0.4679; Figs. 5f, S6A).

## Discussion

We evidenced here inter-individual differences among healthy inbred mice during a decision-making task as already shown during a variant version of the IGT in humans (Bechara et al. 2002) and during the rat gambling task (Rivalan et al. 2009). We confirm and extend our previous report (Pittaras et al. 2013) that healthy C57Bl/6J mice behave differently in a mouse gambling task—MGT—and that behavioral differences rely on neurochemical and brain activation specificities. Solving the MGT requires first an exploration phase in which mice acquire information about each option, then an exploitation phase in which mice use their knowledge about the putative value and risk associated to each option (de Visser et al. 2011c). This knowledge naturally remains imperfect by nature as the response-outcome association is probabilistic. In the exploration phase, mice did not differ from each other. Inter-individual differences emerged only during the exploitation phase. At the end of the MGT, the 54 mice as well as the 24 mice used for immunochemistry, exhibited the same global evolution and inter-individual differences than reported previously (Pittaras et al. 2013). Furthermore, percentage of mice advantageous choices followed a Gaussian type distribution (Fig. S2B), similar to what was observed in a healthy human population during a variant version of the IGT (Bechara et al. 2002). As in humans and rats, a majority of mice (44 %, “average”) preferred advantageous options without neglecting alternative—potentially more risky—choices. Although we cannot rule out the hypothesis that these mice would improve performance if given a couple of more training sessions, we have evidence that their strategies differed from that exhibited by other subgroups the fifth session. We have unpublished data showing that two more sessions of MGT did not change average preferences. A small subgroup of mice (29 %, “safe”) preferred long-term advantageous choices and progressively avoided exploring other options by developing rigid behavior, doing a small number of switches and choosing arms associated with less quinine pellets (even if mice did not eat them). Another small proportion of mice (27 %, “risky”) continued to explore all available options throughout the experiment despite a low probability of getting a reward. Therefore, the MGT allows us to characterize three subgroups of animals regarding their decision-making strategies.

In the elevated plus maze (EPM), risky mice present the same profile as during the MGT, i.e., explorative and non-anxious behavior. This increased exploration of risky or ambiguous options was not associated to a general increase of locomotion, novelty exploration or to a deficit of working memory (Fig. S3). Furthermore, their performance in the MGT was not due to inability to distinguish large from small rewards because risky mice performed normally during the delay-reward task (Fig. 3). In addition, the expected sucrose preference (Ping et al. 2012) was only observed in safe and average groups, but not in the risky group. This apparently surprising result could explain the fact that risky mice were more attracted by novelty exploration than food reward and thus, when subjected to the MGT, continued to visit various arms, including those likely to contain quinine. Altogether, this information suggests that risky mice make choices independently of the probability to get quinine or reward. To that regard, it is noticeable that they did not show more activity in the insular cortex, associated with disgust (Chapman and Anderson 2012). Since food reinforcement is associated to a decreased DA and 5-HT in hippocampus and prefrontal cortex (González-Burgos and Feria-Velasco 2008), the high basal rates of monoamines in the hippocampus (Figs. 5d, h, S6D) of risky mice may prevent them to establish an appropriate action-outcome relationship. In addition, as DA and 5-HT in the hippocampus are necessary for learning and memory (González-Burgos et al. 2008), risky mice may be more prone to explore and learn spatial cues and hence to rely on external information by maintaining exploration phase.

It has been shown that 5-HT plays a key role during top-down control of decision-making (Van den Bos et al. 2013) but some authors found that a low level of extracellular 5-HT is linked with poor performance during decision-making (Heitland et al. 2012; Homberg et al. 2008; Koot et al. 2012; Pittaras et al. 2013; Zeeb et al. 2009) while others did not (Gendle et al. 2010; Homberg et al. 2008; Lage et al. 2011; Macoveanu et al. 2013; Stoltenberg et al. 2011). Here, we observed that risky mice had a high level of 5-HT in the prelimbic (PrL), insular cortices (CIns) and a low level of 5-HT in the orbitofrontal cortex (OFC). We suggest that unbalanced 5-HT levels between the different prefrontal areas—specifically between the OFC and the PrL—lead to more exploratory behavior despite potential risks.

Altogether, these data show that in a healthy mice population, some mice maintained exploration of available options even if associated to uncertain outcomes. A high level of 5-HT, DA and NA in the hippocampus and a low level of 5-HT in the OFC are expected to be markers of this extreme pattern of choices. It has been shown that sensation-seeking, risk-taking and high reactivity to novelty predicts a propensity to initiate cocaine self-administration (Belin et al. 2008, 2011). In addition, level of 5-HT in the OFC plays a key role during top-down control of decision-making (Van den Bos et al. 2013). Regarding these data, risky mice could be good models for vulnerability of addiction or pathological gambling.

Safe mice strongly preferred advantageous options during the MGT. However, they did not choose systematically the arm associated with the larger reward and did not earn more pellets than average mice (Fig. 2b): their apparently more efficient strategy which drives them away from exploration and penalty (quinine pellets), is in fact accompanied by rigid behaviors.

It has been shown that lesion of the OFC or PrL leads to unadapted decision-making (Granon et al. 1994; Rivalan et al. 2011). In addition, it was proposed that the exploration phase requires the activation of the limbic loop and the exploitation phase the activation of the cognitive loop, at the cost of the limbic loop (de Visser et al. 2011a; Koot et al. 2013). This was actually what we observed as safe mice exhibited a hypoactivation of the OFC and of the NAcc at the end of the task (Fig. 4a), two brain areas that are part of the limbic loop. Notably, safe mice exhibited reduced activation of the cognitive loop, specifically the PrL area, as compared to other subgroups. Hypoactivation in safe mice of brain regions involved in the integration of both limbic and cognitive information could explain their important rigidity score at the end of the task. Indeed, OFC, NAcc and PrL brain areas are known to be necessary for flexible behaviors (Boulougouris et al. 2007; Floresco et al. 2009; Mihindou et al. 2013; Young and Shapiro 2009). Moreover, c-fos protein activity in the PrL was negatively correlated with the animal’s performance and rigidity score; therefore we reinforce the fact that a low PrL activity is expected to be a marker of rigid behavior (Floresco et al. 2009). Since safe mice evaluated appropriately the reward value in the sucrose preference test (Fig. 3a) as well as in the delay reward task (Fig. 3d), their choices in the MGT are likely to be guided by penalty avoidance, to the detriment of exploration and flexibility. Low level of risk-taking of safe mice in the EPM reinforces this hypothesis. The monoamine pattern of safe mice is congruent with results obtained in monkeys showing inflexible behaviors associated to regional balance of DA and 5-HT (Groman et al. 2012).

Altogether, these data showed that in a healthy mice population, some mice favor safe strategies to avoid risk and penalty. Hypoactivation of brain areas involved in both limbic and cognitive loops associated with a high level of 5-HT in the OFC combined with low DA level in the CPu are expected to be markers of rigid but safe behavior. It has been shown that anxious subjects performing a risky decision-making task exhibited hypoactivation of the PFC in loss condition (Galván et al. 2014). Moreover, anxiety disorders during adolescence confer increased risk for depression during adulthood (Galván et al. 2014; Kendall et al. 2004; Pine et al. 1998). Although our safe mice did not show general higher level of anxiety in our current experimental conditions, their propensity to prefer conservative and rigid choices could be good traits for vulnerability of anxiety. This prediction would remain to be investigated.

Results of the current study indicate that within inbred healthy mice inter-individual differences exist and can be explained by specific network activity or regional neurochemical markers. As a social group, having different behavioral profiles could be an advantage, if individuals share outcomes. At an individual level, we characterized three different profiles: mice mostly driven by risk avoidance and internal cues, mice which preferred exploration of novel options even those associated to putative risks (these mice were mostly driven by environmental cues), and a third—and larger—subgroup of mice exhibiting balanced choices between the two former extreme profiles therefore showing adaptive decision-making.

In conclusion, we show for the first time that mice subjected to the MGT cope variously to uncertainty and can exhibit extreme patterns of choice and strategy, either rigid or flexible, related to specific monoaminergic and behavioral markers. We expect this work to open the way for the identification of valuable individual markers of vulnerability to psychiatric disorders.

## Electronic supplementary material

Below is the link to the electronic supplementary material.

Supplementary material 1 (DOCX 5821 kb)
