# Supplementary material for: Individual behavioral and neurochemical markers of unadapted decision-making processes in healthy inbred mice
Source: Brain Struct Funct. 2016 Feb 10;221(9):4615–29. doi: 10.1007/s00429-016-1192-2 (PMC5102946; doi:10.1007/s00429-016-1192-2)
Supplement: Supplementary file 1 — Supplementary material 1 (DOCX 5821 kb) [file 429_2016_1192_MOESM1_ESM.docx]

***Supplementary information***

***Supplementary methods***

**Behavioral procedures**

**Habituation in operant chambers before MGT (Pittaras et al. 2013)**

The aim of this experimental part was to habituate mice to be manipulated, to eat pellets in another area than their home cage, to make an effort to get food pellets and to stabilize their weight. Indeed, mice were deprived during all the experiment (4 weeks) and we wanted to maintain it at 85% of their free feeding weight at the end of habituation in operant chambers. Each mouse did one session a day for 10 days. We changed the order of the mice each day. One habituation session lasted 30 minutes.

During a session, the central hole was the only one available. When the mouse did a nose poke one food pellet was distributed. The mouse then had to visit the magazine to eat the pellet and start a new trial.

**Behavioral sub-groups characterization**

***Novelty exploration***

Novelty exploration was measured in a transparent empty Plexiglas cage (50 cm x 30 cm x 30 cm). The light was set at 100 lux in the middle of the cage. We gently put the mouse in the center of the cage and scored different behavioral criteria for 10 min. The cage was cleaned with a solution of water with 10% of alcohol between each mouse. Scoring was made on line. We virtually divided the surface of the floor in eight equal areas and determined mice locomotor activity by scoring the number of visited areas. We also scored the number of rearing (against the wall or not) as exploration.

***Anxiety task (emergence, dark-light)***

*Emergence Task*

Anxiety was measured in a large white openfield (diameter of 110 cm and light set at 100 lux in the middle) connected to a small black start box (20 cm x 20 cm) protected from light by a cover. The experiment lasted 15 min and began with mice placed in the black box. The apparatus was cleaned between each mouse with a solution of water with 10% of alcohol. We recorded on line: the time took by the mouse to emerge in the openfield and the percentage of time spent in it.

*Dark-Light*

The apparatus (Imetronic, Pessac, France) was fully automated and made of two small boxes (20 cm x 20 cm): one black box protected from light by a cover and the other one white and brightly illuminated (1200 Lux). The experiment lasted 10 min. The mouse was gently placed in the corner of the light box and could move freely from one compartment to the other through a dark corridor. The data was computerized automatically (Imetronic, Pessac, France). Behavioral measurements were: initial latency to escape the light box, number of mice passing from the light box to the dark box and the percentage of total time spent in the light box. Apparatus was cleaned with a solution of water with 10% alcohol between each mouse.

***Working memory task (T-maze) (Piérard et al. 2006)***

The behavioral task used to test working memory is based on spontaneous alternation (SA) behavior. Indeed, SA is the innate tendency of rodents to visit arms alternatively at each successive trial. From trial to trial, accurate performance at a given trial (N) requires that subjects are able to discriminate the specific target trial N-1 from the interfering trial N-2. So mice are not only required to keep temporarily in short-term memory specific information but also resetting it over consecutive runs. Working memory is a major component of the sequential alternation performance.

This working memory task was carried out in a T-maze made of opaque grey Plexiglas. Arms were 35 cm long, 10 cm wide and 25 cm high. The start box (10 x 12 cm) was separated from the stem by a vertical sliding door. Vertical sliding doors were also placed at the entrance of each arm. A low-intensity diffuse illumination (10 lux) was provided above the apparatus.

Habituation was done during the first two days: the first day for a habituation to the maze and the second day for a habituation to the sliding door. During the day task, mice began in the starting box and could freely choose one arm. When one arm was chosen we closed the door and let the animal at the end of the arm during 10 sec. Then, we put back the animal in the starting box during 30 sec of Inter Trial Interval (ITI) and a new trial could begin. Between two trials, the T-maze was cleaned using a solution of water with 10% of alcohol, in order to remove any olfactory clues.

**Brain basal monoamine analysis**

Fig. S1 shows localization of our punches used to analyze brain basal monoamines.


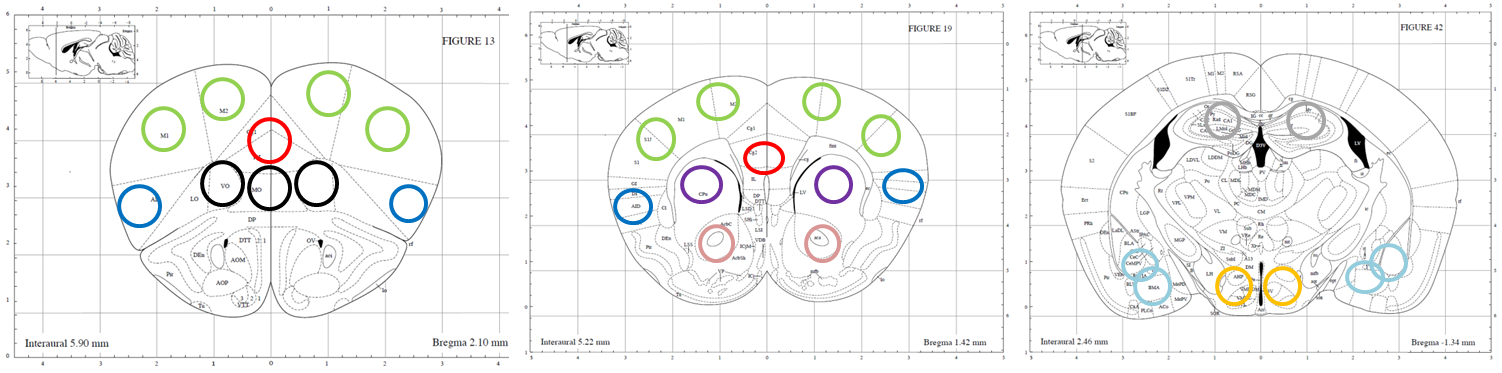


***Supplemental Results***

**Groups’ characterization**

- Overall preferences during the MGT


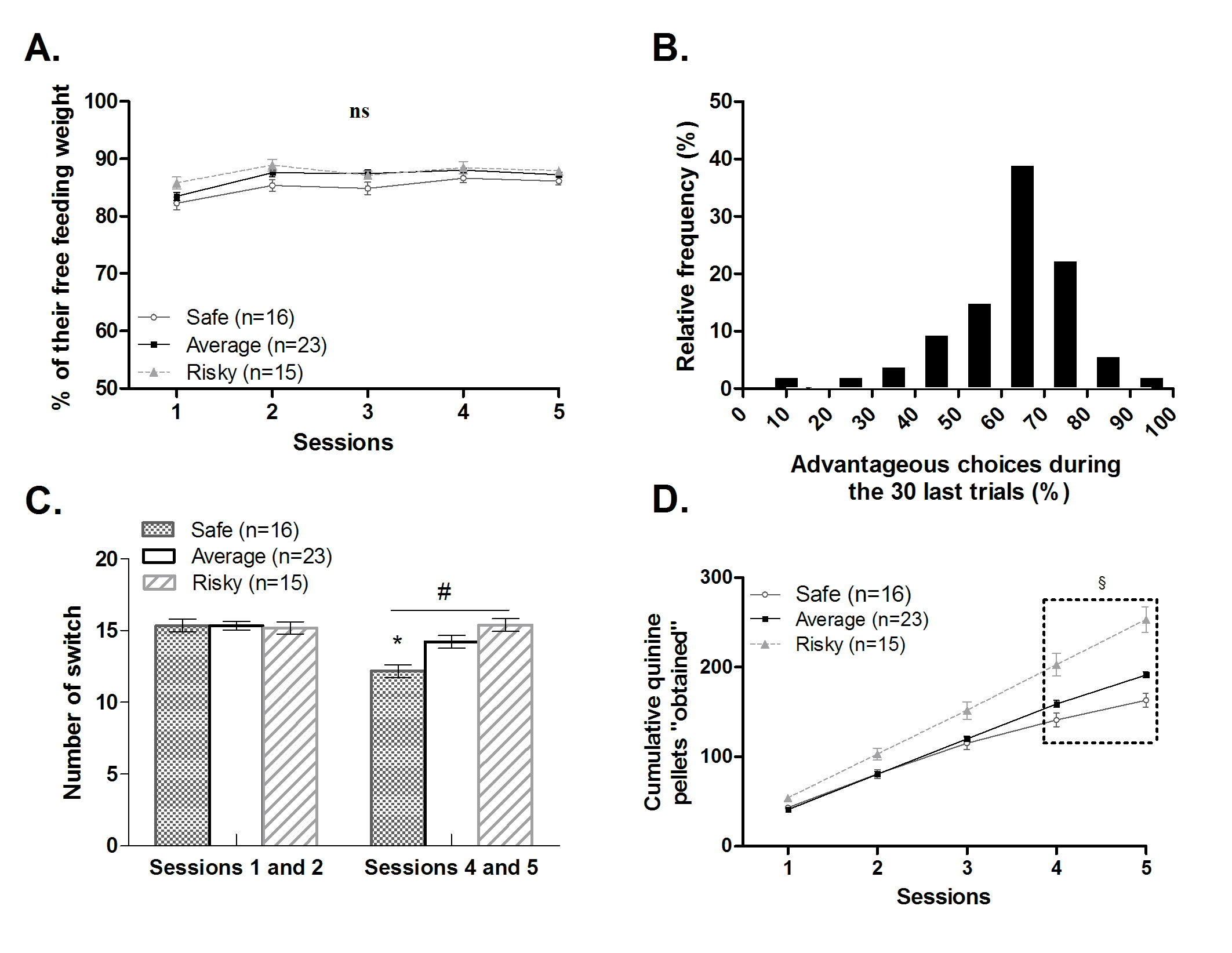


Animal’s weight never differed between groups during the MGT (Fig. S2A; KW: session 1: H=5.974, p=0.0504; session 2: H=5.297, p=0.0707; session 3: H=3.559, p=0.1687; session 4: H=5.309, p=0.0703; session 5: H=3.452, p=0.1780). Fig. S2B represented the repartition of animals regarding their percentage of advantageous choices during the 30 last trials of the MGT. *Safe* animals did significantly less switch during the two last sessions than during the two first sessions (W– Z=-2.973, p=0.0029) and groups differed from each other’s only during the two last sessions (Fig. S2C; KW: session 1 and 2: H=0.114, p=0.9445; session 4 and 5: H=14.042, p=0.0009). As shown in Fig. S2D, cumulative pellets obtained (but not eaten) differed between groups only during the two last sessions (MW– safe *vs.* risky; session 1: U=61.500, p=0.0208; session 2: U=66.500, p=0.0344; session 3: U=59.500, p=0.0168; session 4: U=7.000, p<0.0001; session 5: U =0.000, p<0.0001 ; safe *vs.* average; session 1: U=163.500, p=0.5583; session 2: U=169.500, p=0.6789; session 3: U=152.000, p=0.3609; session 4: U=37.000, p<0.0001; session 5: U=68.500, p=0.001; risky *vs.* average; session 1: U=73.000, p=0.003; session 2: U=104.000, p=0.0408; session 3: U=109.000, p=0.0579; session 4: U=81.500, p=0.0066; session 5: U=23.000, p<0.0001).

- Behavioral characterization of individual differences

As shown in Fig. S3, neither group had troubles concerning locomotor activity (KW: H=2.527, p=0.2826), exploration (KW: H=1.348, p=0.5097) and working memory (KW: H=2.009, p=0.3663). Animals from all groups were able to alternate during the working memory task with an ITI of 30 sec (70 ± 3.5% for *safe* animals, 68.7 ± 3.2 for *average* animals and 77.7 ± 5.5 for *risky* animals) (W task: -safe- Z=6.008, p=0.0003; -average- Z=5.960, p<0.0001; -risky- Z=5.330, p=0.0007). Because the proportion of mice (around 40%) which didn’t alternate during the working memory task with an ITI of 30 sec was the same for each group (*safe*, *average* and *risky*) we excluded this mice for the study (Chauveau et al. 2014). As a result, differences observed during the MGT were not linked with hyperactivity or a working memory deficit.

##
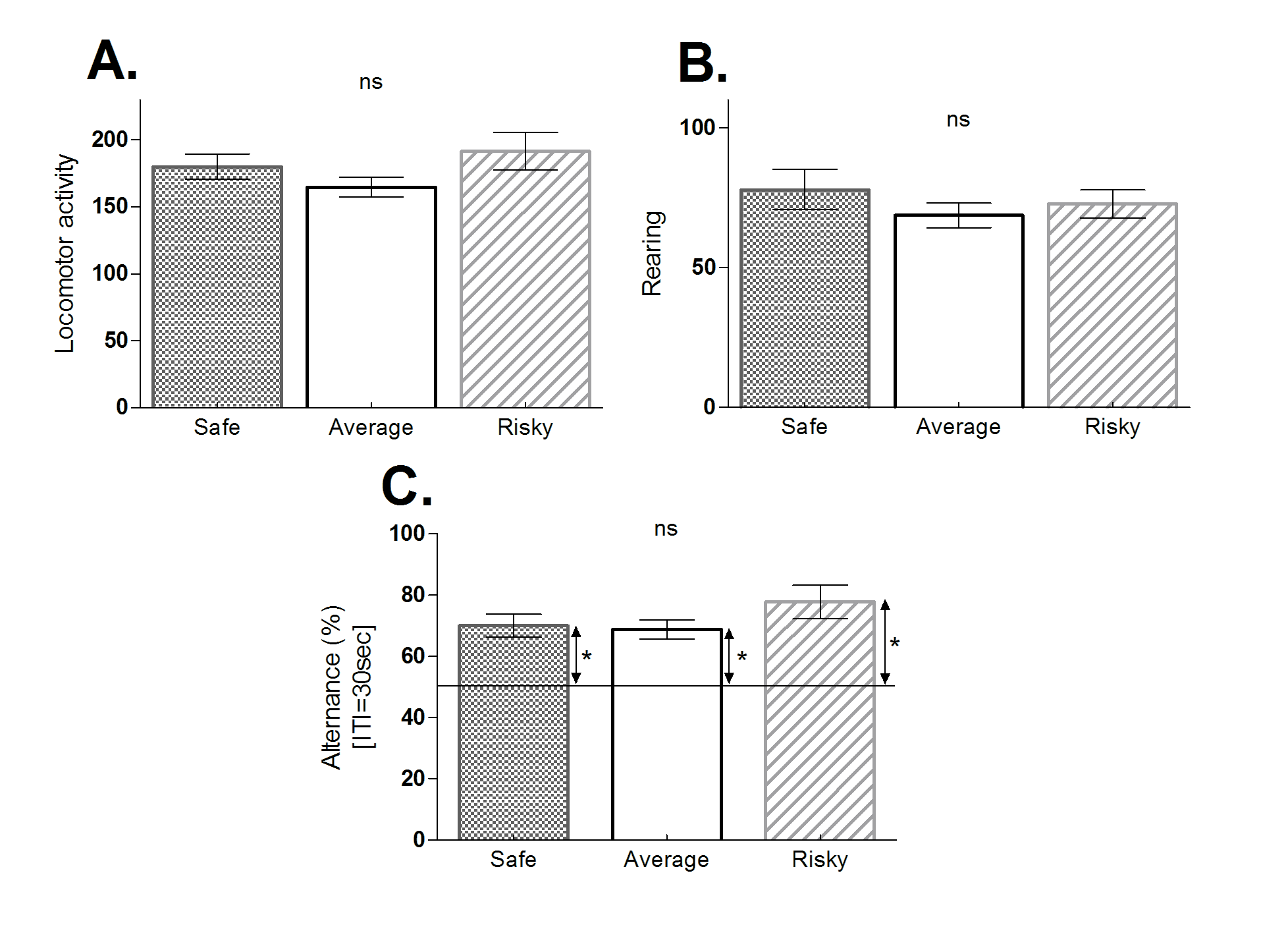


Groups differed from each other regarding the latency to escape during the dark light task (KW: H=8.528, p=0.0141). *Safe* animals took less time to escape than *average* animals (3.1 ± 0.6sec for *safe* animals and 5.9 ± 1.1sec for *average* animals) (MW: U=81.500, p=0.0034). Whereas all animals took around 90 sec to emerge in the openfield (KW: Z=2.637, p=0.2676). All animals spent between 35% and 45% of the experiment time in the light box during the dark light task and in the openfield during the emergence task (KW: -emergence task- H=0.623, p=0.7324; -dark light task- H=1.452, p=0.4837). No significant differences existed between groups regarding the number of passages from the light box to the dark box during the dark light task (22.3 ± 2.3 for *safe* animals, 17.3 ± 1.8 for *average* animals and 19.3 ± 1.7 for *risky* animals) (KW: H=3.323, p=0.1899; Fig. S4). These data indicated that *safe* animals seemed to be more anxious but not in all behavior tasks.


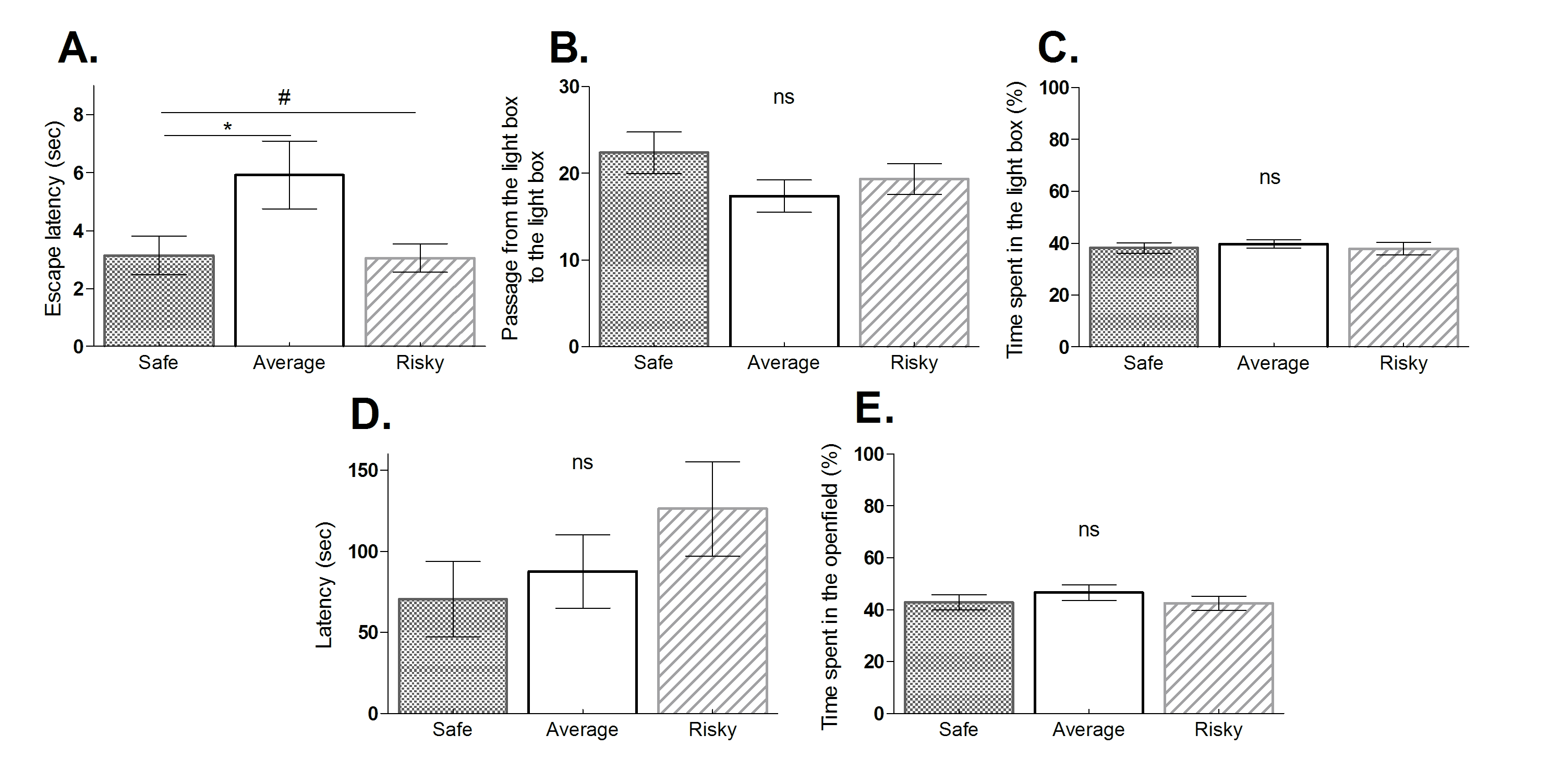


- Neurobiological characterization of individual differences

Initially animals equally chose options, except *risky* animals during the first session (60 ± 4% of advantageous choices) (W test – session 1: Z=-2.023, p=0.0431; session 2: Z=-0.405, p=0.6858; session 3: Z=-0.000, p>0.999; session 4: Z=-0.210, p=0.8339; session 5: Z=-0.000, p>0.999). Over time, 3 groups emerged: *safe*, *average* and *risky* animals (84 ± 1.4% *vs.* 67.3 ± 2.3% vs 49.3 ± 7.2% the last session) (MW– safe *vs.* risky: U prime=30.000, p=0.0062; safe *vs.* average: U Prime=65.000, p=0.0014; risky *vs.* average: U=13.500, p=0.0253). As observed before, *average* (66.5 ± 4.4% and 67.31 ± 2.3% of advantageous options) and *safe* (65 ± 3.1%, 72 ± 6.2% and 84 ± 1.4) animals progressively chose advantageous options (W test average – session 1: Z=-0.594, p=0.5525; session 2: Z=-1.490, p=0.1361; session 3: Z=-1.607, p=0.1080; session 4: Z=-2.795, p=0.0052; session 5: Z=-3.059, p=0.0022 - W test safe – session 1: Z=-0.183, p=0.8581; session 2: Z=-0.405, p=0.6858; session 3: Z=-1.826, p=0.0679; session 4: Z=-2.023, p=0.0431; session 5: Z=-2.023, p=0.0431).

As the 54 mice, 24 mice progressively chose more long-term advantageous options (from 53.7 ± 3.1% of advantageous choices to 66.6 ± 3.2%) and inter-individuals differences emerged (Fig. S5).

##
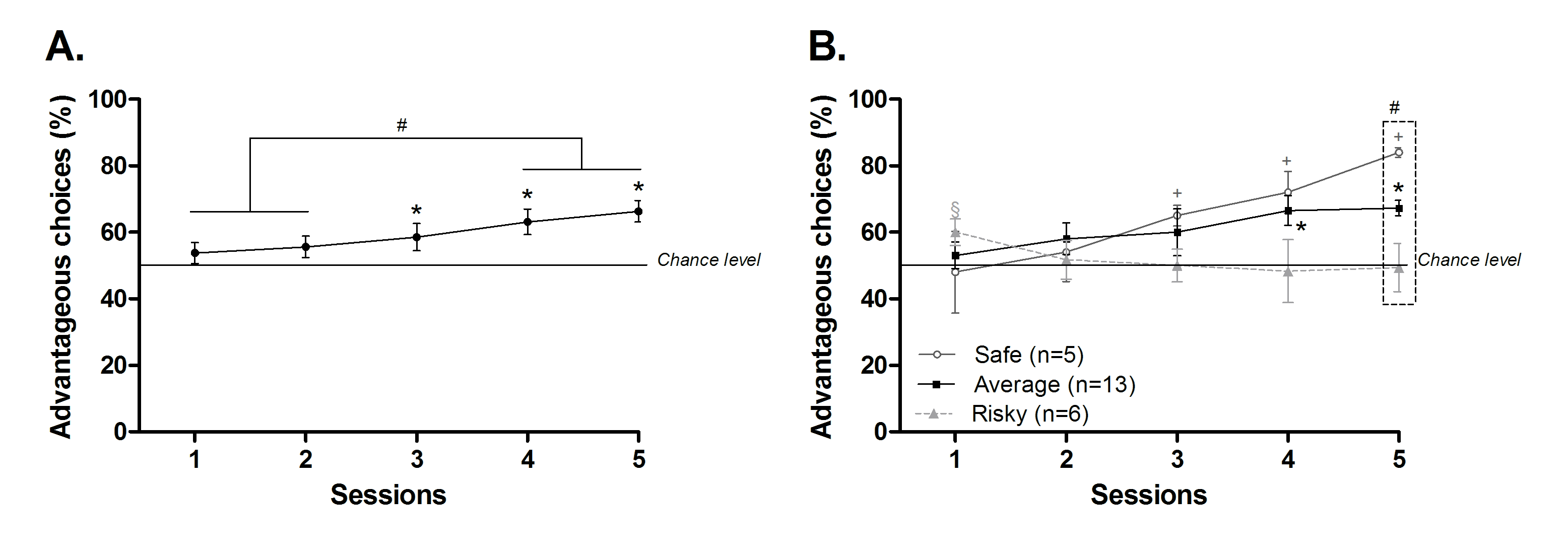


- Basal rate of cerebral monoamines for the 3 MGT groups

No differences existed between groups regarding dopamine (DA) level in the orbitofrontal cortex (OFC) (KW: H=3.556, p=1690; Fig. S6A).

*Safe* mice had a lower level of noradrenaline (NA) in the OFC than the other groups (KW: H=12.282, p=0.0022; MW– safe *vs.* risky: U=74.500, p=0.001; safe *vs.* average: U=132.000, p=0.0044; risky *vs.* average: U=132.000, p=0.7725; Fig. S6B) and a higher one in the nucleus accumbens (NAcc) (KW: H=13.896, p=0.005; MW– safe *vs.* risky: U=36.000, p=0.0016; safe *vs.* average: U=114.000, p=0.0015; risky *vs.* average: U=114.000, p=0.3629; Fig. S6C).

*Risky* mice had a more important level of NA in the hippocampus than the other groups (KW: H=14.103, p=0.0009; MW– safe *vs.* risky: U=32.000, p=0.0009; safe *vs.* average: U=68.500, p=0.2325; risky *vs.* average: U=68.500, p=0.0124; Fig. S6D).


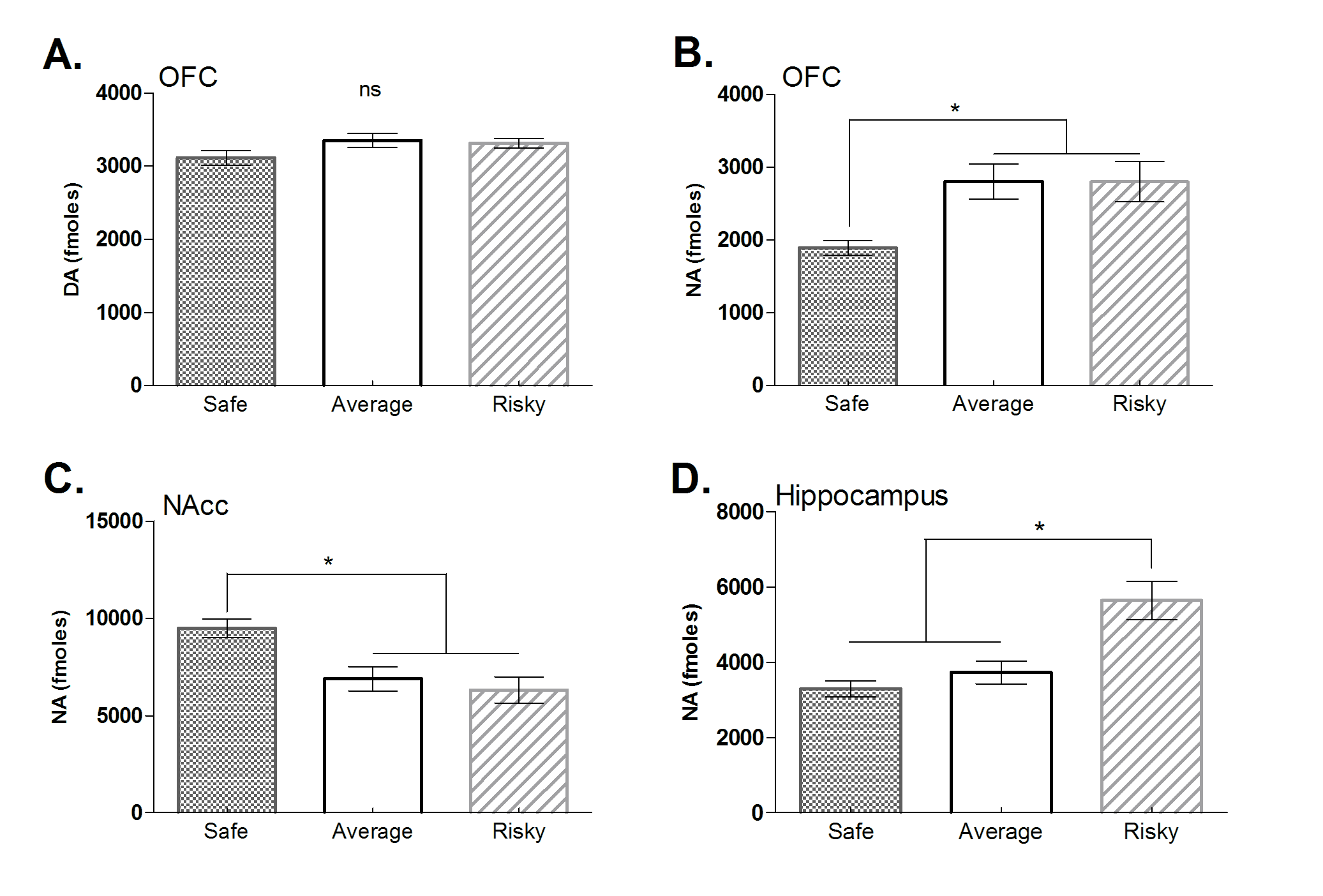


Fig. S1 : Localization of the punches during section sampling. Motor cortex (green circle), orbitofrontal cortex (black circle), limbic cortex (red circle), insular cortex (blue circle), nucleus accumbens (pink circles), dorsal striatum (purple circles), hypothalamus (yellow circle), amygdala (blue circle) and hippocampus (grey circles) were removed from mice brains.

Fig. S2: A. Animals never differed from each other regarding their weight during all MGT sessions (KW, ns p>0.5). B. Animal’s repartition regarding their percentage of advantageous choices during the 30 last trials of the MGT C. Mean of the number of switch during the two first and last sessions of the MGT for *safe* (n=16), *average* (n=23) and *risky* (n=15) animals. *Safe* animals did less switch at the end of the MGT (*, W p<0.05) and the 3 subgroups differ from each other only at the end of the MGT (#, KW p<0.05). D. Quinine pellets obtained (but not eaten) during the MGT for *safe* (n=16), *average* (n=23) and *risky* (n=15) animals. During the two last sessions, *safe* mice obtained les quinine pellets than *average* mice which obtained less pellets than *risky* mice (§, KW p<0.05).

Fig. S3: *Safe* (n=16), *average* (n=23) and *risky* (n=15) animals didn't differ from each other regarding locomotor activity (A.) and exploration (B.). Likewise *Safe* (n=9), *average* (n=15) and *risky* (n=9) animals didn't differ from each other regarding working memory (C.) and all animals preferred sucrose (*, W p<0.05). ns represented no significant differences between groups (KW).

Fig. S4: Characterization of groups regarding anxiety. Escape latency the light box (A.), number of moves from the light box to the dark box (B.) and time spent in the light box were analyzed during the dark light task (C.) (MW, * p<0.05). Latency to enter (D.) and time spent (E.) in the openfield were analyzed during the emergence task. ns represented no significant differences and # p<0.05 a significant difference between the *safe* (n=16), *average* (n=23) and *risky* (n=15) animals (KW).

Fig. S5: A. Global performance during the MGT. # represents difference between sessions and * represents difference from chance level (50%) (W, p<0.05). B. Evolution according to sessions for each sub-groups: *safe* (n=5), *average* (n=13) and *risky* (n=6). + represents difference from chance for *safe* animals (W, p<0.05), * represents difference from chance for *average* animals (W, p<0.05) and § represents difference from the chance for *risky* animals (W, p<0.05). # represents differences between the three groups (MW, # p<0.05).

Fig. S6 : Basal rates of monoamines for *safe* (n=16), *average* (n=20) and *risky* (n=14) animals. Basal rates of dopamine (DA) in the Orbitofrontal Cortex (OFC) (A.) and basal rates of noradrenaline (NA) in the OFC (B.), nucleus accumbens (NAcc) (C.) and the hippocampus (D.). Results are represented as mean ± s.e.m. * p<0.05 represents a significant difference between groups (MW) and ns represented no significant difference between groups (MW). No difference existed between groups regarding dopamine level in the OFC (A.). *Safe* mice had a low level of noradrenaline in the OFC (B.) but a high level in the NAcc (C.). Risky mice had a higher level of NA in the hippocampus (MW, * p<0.05).
